# Traumatic rupture of an isolated splenic hydatid cyst: a rare and challenging surgical emergency

**DOI:** 10.1093/jscr/rjag317

**Published:** 2026-05-06

**Authors:** Hazem Ahmad AlShikh, Amal Ali Al Aeid, Beshr Mosa Basha, Mariam M Hussain, Rana Ahmed Abdelnaby, Fadhel Alherz

**Affiliations:** Department of General Surgery, Dr Sulaiman AlHabib Medical Group, King Salman Bin Abdulaziz Rd, Al Bandariyah, Al Khobar 24423, Saudi Arabia; Department of General Surgery, Dr Sulaiman AlHabib Medical Group, King Salman Bin Abdulaziz Rd, Al Bandariyah, Al Khobar 24423, Saudi Arabia; Department of General Surgery, Dr Sulaiman AlHabib Medical Group, King Salman Bin Abdulaziz Rd, Al Bandariyah, Al Khobar 24423, Saudi Arabia; Department of General Surgery, Dr Sulaiman AlHabib Medical Group, King Salman Bin Abdulaziz Rd, Al Bandariyah, Al Khobar 24423, Saudi Arabia; Department of General Surgery, Dr Sulaiman AlHabib Medical Group, King Salman Bin Abdulaziz Rd, Al Bandariyah, Al Khobar 24423, Saudi Arabia; Department of General Surgery, Dr Sulaiman AlHabib Medical Group, King Salman Bin Abdulaziz Rd, Al Bandariyah, Al Khobar 24423, Saudi Arabia

**Keywords:** trauma-induced rupture, splenic hydatid cyst, echinococcosis, acute abdomen, surgical management

## Abstract

Isolated splenic hydatidosis is a rare manifestation of *Echinococcus granulosus* infection, accounting for ~0.5%–4% of reported cases. Although often asymptomatic, cyst rupture is an uncommon but severe complication that may result in life-threatening systemic reactions, frequently precipitated by trauma. A 65-year-old Saudi male with type 2 diabetes mellitus and hypertension presented with acute epigastric pain, nausea, vomiting, and low-grade fever one week after minor trauma. He was hypotensive with a tender, non-peritonitic abdomen, and elevated inflammatory markers. Contrast-enhanced computed tomography demonstrated a large ruptured splenic hydatid cyst with intraperitoneal fluid. Emergency laparotomy with total splenectomy was performed, and histopathology confirmed the diagnosis. Recovery was uneventful, and albendazole therapy was initiated. Minor trauma may precipitate rupture of an unrecognized splenic hydatid cyst, and early imaging with prompt surgical management is critical to prevent life-threatening complications, particularly in endemic regions.

## Introduction

Isolated splenic hydatidosis is a rare manifestation of hydatid disease, accounting for ~0.5%–4% of reported cases, and typically presents with nonspecific symptoms such as abdominal pain, nausea, vomiting, fever, and splenomegaly on examination [1]. It may mimic other splenic cystic lesions, including congenital cysts, abscesses, or neoplasms [2], and can remain asymptomatic until complications arise, leading to incidental discovery [3]. Reported complications include compression of adjacent structures, such as splenic vein compression causing segmental portal hypertension or renal arterial compression resulting in secondary systemic hypertension. More critically, spontaneous or trauma-related rupture may lead to intraperitoneal dissemination and severe hypersensitivity reactions or anaphylactic shock due to spillage of highly antigenic cyst fluid, which can be life-threatening [4, 5]. Only a limited number of ruptured isolated splenic hydatid cysts have been described in recent decades, making early recognition and timely management essential. This report presents a rare traumatic rupture and highlights its clinical and surgical implications.

## Case presentation

A 65-year-old Saudi male from the southern region of Saudi Arabia, a non-smoker with type 2 diabetes mellitus and hypertension, had a history of ischemic stroke 11 years earlier with mild residual left-sided weakness and previous right inguinal hernia repair. He presented to the emergency department with a 3-day history of acute epigastric pain associated with nausea, vomiting, intermittent low-grade fever with chills, and marked fatigue. One week prior to presentation, he had sustained minor trauma after falling onto his left side without fractures. Following the traumatic event, his pain gradually worsened over several days. He initially managed his symptoms at home with analgesics; however, his condition progressively deteriorated, with the development of low-grade fever and loss of appetite, ultimately prompting presentation to the emergency department. He also reported a history of mild, intermittent left upper quadrant pain for ~10 months prior to presentation. On examination, he was conscious and oriented with a Glasgow Coma Scale score of 15/15. Vital signs showed hypotension (92/51 mmHg), with mild tachycardia (heart rate 105 bpm), respiratory rate of 22–24 breaths/min, and temperature of 37.6°C. Oxygen saturation was 92% on room air. Venous blood gas analysis revealed an elevated lactate level of 2.9 mmol/l. The patient responded well to initial fluid resuscitation, with subsequent improvement in blood pressure, and did not require vasopressor support. The abdomen was soft and non-distended, with marked tenderness in the left upper quadrant and mild generalized abdominal tenderness, without guarding or rigidity. Rebound tenderness was not clearly elicited.

**Figure 1:**
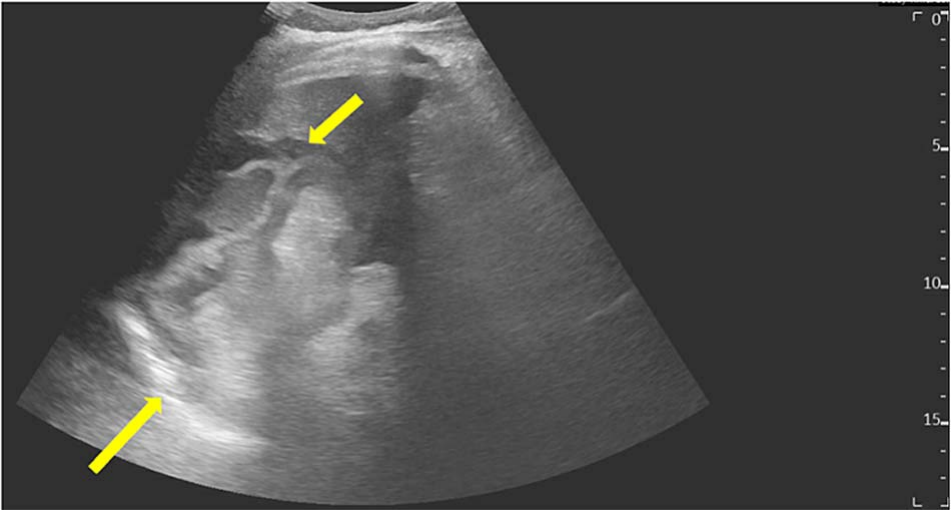
Abdominal ultrasound showing a spleen of average size with preserved contour and echotexture, associated with minimal intraperitoneal free fluid. An ill-defined structure is seen adjacent to the spleen beneath the left hemidiaphragm.

**Figure 2:**
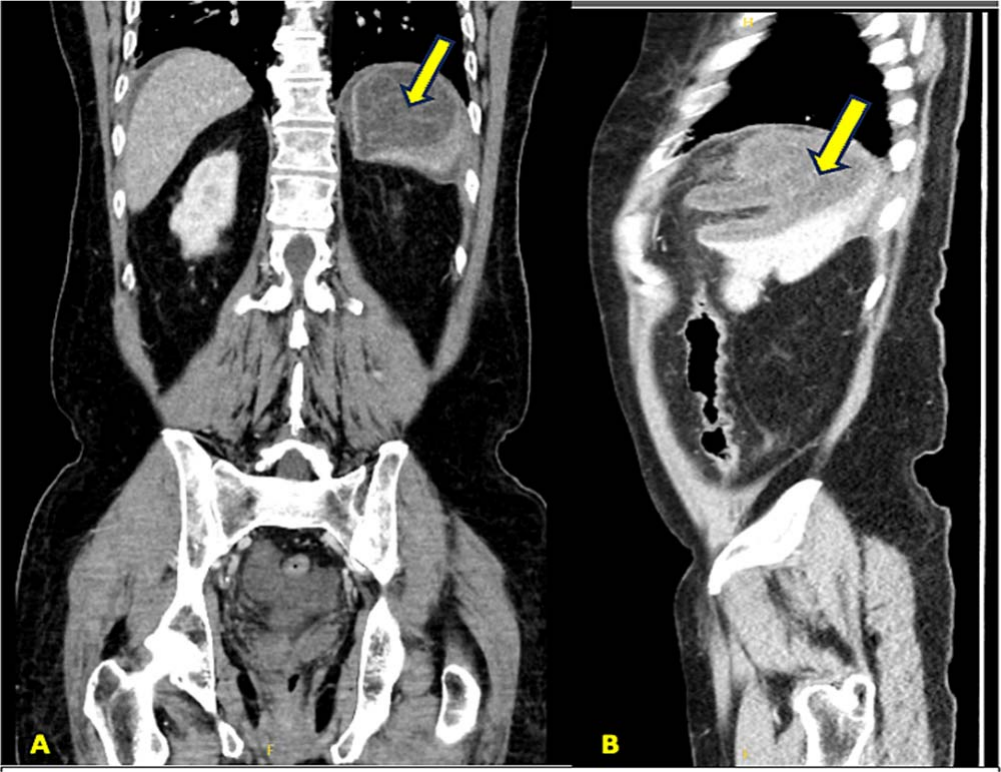
Contrast-enhanced abdominal CT images showing a large irregular cystic lesion measuring 8.0 × 15.0 × 7.3 cm in the left subdiaphragmatic region. (A) Coronal view and (B) sagittal view demonstrating an internal floating membrane. The lesion is inseparable from the splenic parenchyma, consistent with a splenic origin.

**Figure 3:**
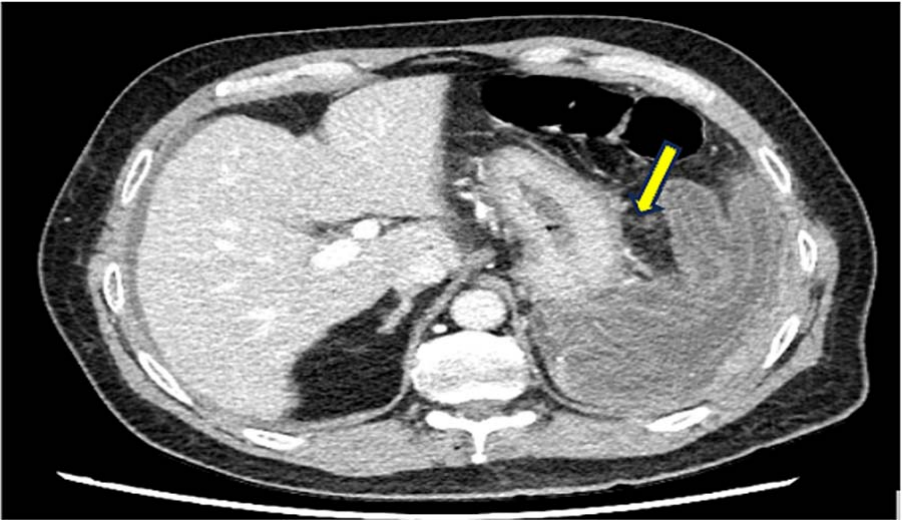
Axial contrast-enhanced CT image demonstrating inflammatory fat stranding surrounding the splenic lesion, with associated free intraperitoneal fluid.

Laboratory investigations revealed leukocytosis (12.8 × 10^3^/μl) with neutrophilia (11.6 × 10^3^/μl), elevated C-reactive protein (168 mg/l), and ferritin of 459 μg/l. Abdominal ultrasonography was performed after initial resuscitation, demonstrating a spleen of normal size with minimal intraperitoneal free fluid and an ill-defined structure in the left upper abdomen (Fig. 1). Given these findings, and to exclude other potential acute surgical pathologies that could explain the patient’s presentation, further evaluation with contrast-enhanced computed tomography was undertaken following hemodynamic stabilization. Computed tomography (CT) revealed a large irregular cystic lesion (8.0 × 15.0 × 7.3 cm) in the left subdiaphragmatic region containing a characteristic internal floating membrane and inseparable from the splenic parenchyma, with surrounding inflammatory fat stranding and minimal free fluid—findings consistent with a ruptured splenic hydatid cyst (Figs 2 and 3). Given the diagnosis of a ruptured splenic hydatid cyst associated with hemodynamic instability, the patient underwent emergency exploratory laparotomy.

**Figure 4:**
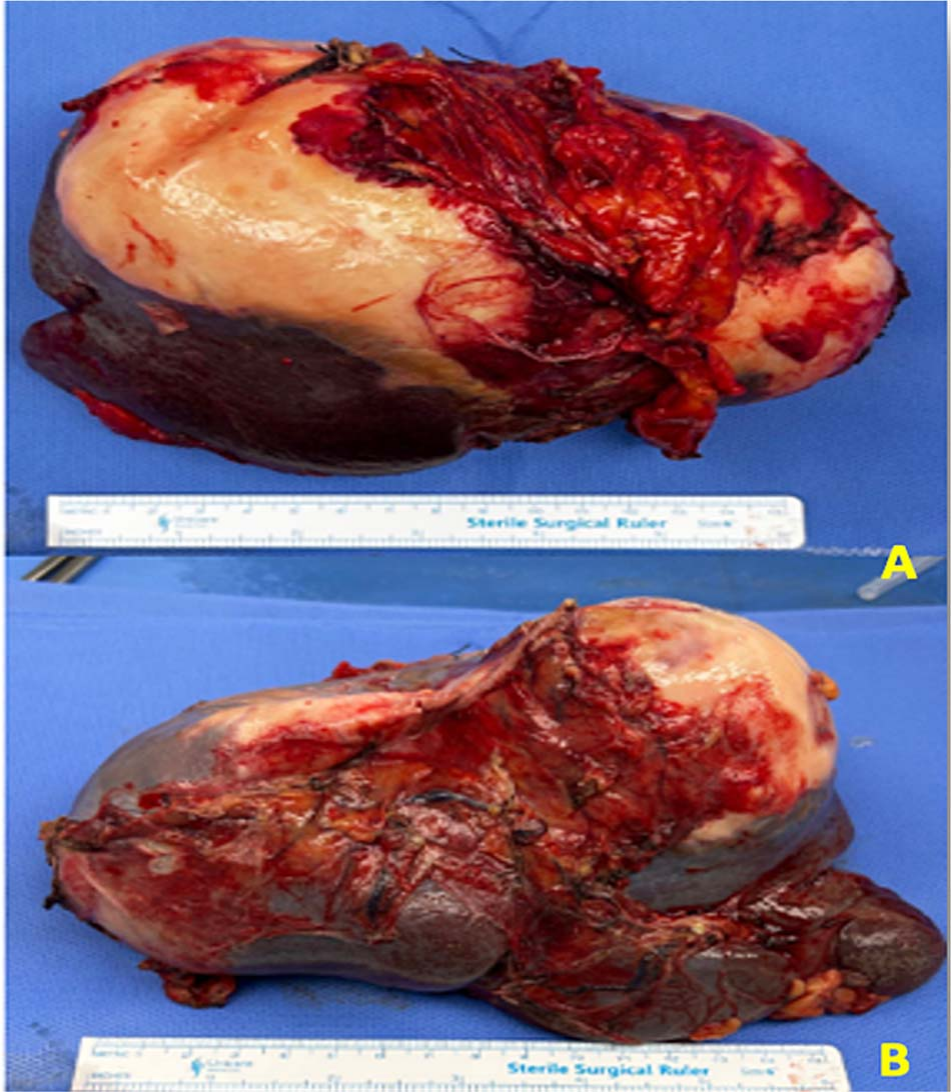
Gross pathological specimen of the spleen demonstrating a well-defined cystic lesion with a thick laminated wall and a whitish, pearly appearance. (A and B) Different views of the specimen.

Intraoperatively, there was localized, contained turbid fluid in the left upper quadrant, without evidence of generalized peritoneal contamination or significant hemoperitoneum. No daughter cyst dissemination was observed, and the findings were consistent with a limited or contained rupture. Peritoneal lavage was performed using ~2 l of hypertonic saline as a scolicidal agent, with careful handling of the cyst and controlled evacuation to minimize spillage and prevent secondary echinococcosis. The spleen showed no active bleeding; however, the cyst was inseparable from the splenic hilum, with dense adhesions involving the splenic vessels. A small capsular disruption was noted at the splenophrenic ligament attachment. In addition, the cyst was adherent to the diaphragm, with partial-thickness involvement of the outer muscular layer. A defect measuring ~3 cm was identified and managed with limited excision and primary repair. There was no evidence of pleural breach or intrathoracic contamination, and a chest tube was not required. Intraoperative chest X-ray was unremarkable. Given these findings, total splenectomy was performed. Gross pathological examination confirmed a splenic hydatid cyst (Fig. 4A and B). Histopathological examination subsequently confirmed the diagnosis. Postoperatively, the patient improved in the intensive care unit and had an uneventful recovery. He was discharged on postoperative day nine with albendazole 400 mg twice daily for 30 days following multidisciplinary discussion with the infectious diseases team. On follow-up, therapy was extended for an additional 30 days, with a plan for further reassessment.

During outpatient follow-up over the first 2 months, he remained clinically well, with no complaints and appropriate wound healing, and there was no clinical evidence of recurrence. The patient was advised to continue regular follow-up, including clinical assessment, imaging, and serological evaluation as part of routine postoperative surveillance. He also received post-splenectomy vaccinations 2 weeks after surgery in accordance with infectious diseases recommendations.

## Discussion

Cystic echinococcosis most commonly affects the liver (≈70%) and lungs (≈25%), whereas splenic involvement is rare, occurring in only 0.5%–4% of reported cases [1]. These cysts may enlarge to 5–10 cm during early growth and remain asymptomatic for years [3]. With progressive enlargement, symptoms may arise from mass effect or local inflammation, typically presenting with abdominal pain, fever, or splenomegaly, while gastrointestinal or diaphragmatic compression may occur in advanced disease [5]. The absence of specific clinical features often delays diagnosis, and many cases are detected incidentally. Intraperitoneal rupture is an uncommon but potentially life-threatening complication, reported in ~1.7% of hydatid disease cases, and may follow minor trauma or spontaneous increases in intracystic pressure, resulting in acute abdominal pain, hemodynamic instability, allergic manifestations, or anaphylactic shock [6–8]. In contrast, our patient presented with hemodynamic instability without overt allergic features, highlighting the variable and occasionally atypical presentation of ruptured splenic hydatid disease. This may be explained by the contained nature of the rupture, with limited intraperitoneal spillage of antigenic cyst contents rather than free rupture into the peritoneal cavity. Intraoperative findings of localized, contained fluid without generalized peritoneal contamination or daughter cyst dissemination support this mechanism. The delayed clinical course further suggests a gradual leak rather than abrupt complete rupture, which may account for both the absence of acute peritoneal irritation and the lack of allergic manifestations. Furthermore, the patient’s history of chronic mild abdominal pain supports the presence of a pre-existing cyst, possibly in a less active stage. Trauma may have acted as a precipitating factor leading to progressive cyst disruption and delayed clinical deterioration. However, spontaneous rupture cannot be entirely excluded, and the temporal association with trauma should be interpreted cautiously. In suspected intraperitoneal rupture, ultrasonography is typically the initial diagnostic modality, particularly in hemodynamically unstable patients, with a reported sensitivity of ~85%. In contrast, contrast-enhanced computed tomography in stable patients provides near-complete diagnostic accuracy and is pivotal for confirming rupture and guiding surgical management [7, 8]. In our case, ultrasound offered initial diagnostic orientation, while hemodynamic stability and prompt CT access enabled definitive confirmation and timely operative planning. Emergency splenectomy remains the standard treatment for ruptured splenic hydatid cysts, providing definitive control of contamination and preventing life-threatening complications [5, 6, 8]. Spleen-preserving strategies have been described in carefully selected hemodynamically stable patients to reduce the risk of overwhelming post-splenectomy infection, with some reports demonstrating no significant difference in recurrence compared with total splenectomy [6]. In our case, urgent total splenectomy was required due to diaphragmatic involvement and hemodynamic compromise. Additionally, spleen preservation was not feasible because of hilar involvement and dense adhesions to the splenic vessels, which increased the risk of vascular injury and incomplete source control. Therefore, total splenectomy was considered the most appropriate and definitive surgical management. Postoperatively, albendazole therapy is recommended to reduce the risk of secondary echinococcosis and recurrence and is often continued for several months following surgery [6, 8]. Meticulous follow-up is essential for early detection and management of hydatid cyst recurrence. Following intraperitoneal rupture of cystic echinococcosis, reported recurrence rates range from 6.7% to 28%, as described by Beyrouti *et al*., Derici *et al*., and Kurt *et al*. [9–11]. Postoperative surveillance typically includes clinical assessment, imaging—most commonly ultrasonography—and serological evaluation. Some protocols recommend follow-up imaging at ~6 months after surgery, with continued long-term surveillance to detect recurrence or secondary dissemination [12]. Although isolated splenic hydatid cysts have been previously reported, rupture precipitated by minor trauma with diaphragmatic involvement and hemodynamic compromise remains distinctly uncommon. This case highlights the importance of early imaging, timely surgical intervention, and coordinated multidisciplinary management to prevent life-threatening outcomes, particularly in endemic regions.

## Conclusion

Splenic hydatid disease is a rare manifestation of cystic echinococcosis, even in endemic regions. This case demonstrates how minor trauma may precipitate rupture of an unrecognized splenic hydatid cyst, resulting in life-threatening clinical deterioration in an elderly patient with comorbidities. Early computed tomography and prompt surgical intervention were essential in preventing complications such as peritoneal dissemination and anaphylaxis. Clinicians in endemic areas should maintain a high index of suspicion in acute abdominal presentations after trauma. Early imaging, appropriate surgery, and tailored antiparasitic therapy remain critical to improving outcomes in this uncommon but serious condition.
